# Targeting FGF23 to Ameliorate Anemia in Chronic Kidney Disease

**DOI:** 10.3390/ijms27198618

**Published:** 2026-09-26

**Authors:** Karen Cecilia Valdés-Díaz, Raquel María García-Sáez, Casimiro Valle, Ana Isabel Torralbo-Romero, Daniel Jurado-Montoya, Antonio Rivas-Domínguez, Teresa Obrero, María José Jiménez, Fátima Guerrero, Rodrigo López-Baltanás, Rafael Serrano-Berzosa, Miguel Mendiburu-Quintana, Antonio Canalejo, Mariano Rodríguez, Juan Rafael Muñoz-Castañeda, Alejandro Martín-Malo, María Encarnación Rodríguez-Ortiz

**Affiliations:** 1 Maimonides Biomedical Research Institute of Cordoba (IMIBIC), University Hospital Reina Sofia, University of Cordoba, 14004 Cordoba, Spainraquel.garcia@imibic.org (R.M.G.-S.);; 2 Health Results-Oriented Cooperative Research Network RICORS2040 RD24/0004/0004 (Kidney Disease), Institute of Health Carlos III, 28029 Madrid, Spain; 3 Nephrology Service, Reina Sofia University Hospital, Maimonides Institute for Research in Biomedicine of Cordoba (IMIBIC), University of Cordoba, 14004 Cordoba, Spainamartinma@senefro.org (A.M.-M.); 4 Department of Integrated Sciences, Research Center on Natural Resources, Health, and Environment (RENSMA), University of Huelva, 21007 Huelva, Spain

**Keywords:** chronic kidney disease, anemia, phosphorus, FGF23, HIF1Alpha

## Abstract

The progression of chronic kidney disease (CKD) entails the development of complications, such as anemia and disturbances in mineral homeostasis, among others, and both of them have been shown to be closely related. In fact, high phosphorus (P) and excessive production of fibroblast growth factor 23 (FGF23) are hallmarks of the mineral alterations found in CKD, both of which have been associated with the development of anemia. The objective of this study was to evaluate whether the modulation of P load may impact the severity of CKD-related anemia as well as to separate the effects of FGF23 from those of P by the administration of an AntiFGF23 antibody while also investigating the involvement of hypoxia-inducible factor (HIF) signaling in these actions. To address this objective, we used a CKD model based on 5/6 nephrectomy (Nx). In a first experiment, CKD rats received diets with 0.2, 0.6, or 1.2% of P for 4 weeks. In a second study, Nx rats fed a 1.2% P diet were treated with an AntiFGF23 antibody for the same period. In an additional experiment, the effect of the exogenous administration of FGF23 and AntiFGF23 was evaluated. In all the experiments, we analyzed the hematological status, parameters of renal function and mineral metabolism, and HIF signaling. In CKD, the increase in P load was associated with an expected progressive elevation in FGF23 and an aggravation of anemia. In a situation of uremia with high P load, the neutralization of FGF23 restored hematological alterations while producing a significant inhibition in renal HIF1Alpha. In the absence of renal damage, the administration of recombinant FGF23 produced a decrease in serum iron and reticulocytes without changes in HIF signaling. In conclusion, progressive and concomitant increases in P and FGF23 produce an aggravation of anemia of renal origin. In this setting, the normalization of hematological parameters induced by FGF23 blockade is associated with the inhibition of renal HIF signaling. Thus, the implementation of FGF23-targeting therapies might constitute an additional strategy for the clinical management of CKD-related anemia.

## 1. Introduction

Anemia is a frequent complication in chronic kidney disease (CKD). Anemia of renal origin is considered to have a multifactorial etiology, involving impairment in erythropoiesis, reduced renal erythropoietin production, and dysregulation of iron metabolism, among other factors [1].

On the other hand, the onset and progression of chronic kidney disease (CKD) entail the development of disturbances in mineral homeostasis. Among them, renal dysfunction determines a positive balance of phosphorus (P). In early CKD, adaptive mechanisms are aimed at counteracting P retention. In fact, as renal disease progresses, there is a progressive increase in the production of fibroblast growth factor 23 (FGF23) to promote phosphaturia and prevent the increase in plasma P [2]. Nevertheless, this mechanism eventually fails, and hyperphosphatemia develops. With progressive loss of functional nephron mass, the P excretion required per remaining nephron must increase to maintain P balance. Once nephron loss exceeds a critical threshold, typically an estimated glomerular filtration rate below 30 mL/min/1.73 m^2^, the remaining nephrons reach the limit of their excretory capacity, and further elevations in circulating FGF23 can no longer compensate for the reduced renal functional mass [3,4]. Consequently, hyperphosphatemia develops not only because FGF23 signaling itself becomes ineffective, but also because too few functioning nephrons remain to maintain adequate P excretion despite markedly elevated FGF23 levels.

Anemia and CKD exhibit a bidirectional relationship. Thus, the severity of anemia increases as CKD progresses, while the effective management of anemia may slow down the progression of renal dysfunction [5,6]. Although the development of anemia has a multifactorial nature, disturbances in P and FGF23 are relevant components of CKD pathophysiology and have been involved in the progression of renal dysfunction. In addition to their role in CKD pathophysiology, disturbances in P and FGF23 have been associated with impairment in iron metabolism and development of anemia in patients with CKD. Therefore, both elements may represent relevant links between CKD progression and the development of renal anemia.

The relationship between P and anemia has been evidenced in the clinical setting, showing an inverse relationship between hyperphosphatemia and low hemoglobin [7,8,9]. However, despite these findings, it remains to be known to what extent variations in the dietary P load may modulate the degree of anemia and whether dietary P restriction may have an impact on its onset and progression.

On the other hand, FGF23 has also been suggested to have a potential role in the development of anemia in the context of renal dysfunction. Hence, pediatric CKD patients with anemia exhibit higher FGF23 levels compared with patients without a diagnosis of anemia [10]. In adult patients, similar associations have been found with prevalent anemia, change in hemoglobin over time, and onset of anemia [11]. Furthermore, there is evidence that the inhibition of FGF23 signaling acutely rescues renal anemia [12], although it is unknown whether this effect can be reversed over time, as well as the molecular mechanisms involved in this regulatory process. Furthermore, the implications of the presence of high FGF23 levels in a situation where kidney function is normal have not been determined yet.

On the other hand, hypoxia-inducible factors (HIFs) are heterodimeric transcription factors containing an oxygen-regulated alpha subunit. Under normoxic conditions, HIF-alpha subunits are hydroxylated by prolil-hydroxilase enzymes in an oxygen-dependent manner, subsequently undergoing proteasomal degradation. Under hypoxic conditions, prolil-hydroxilases activity is inhibited, and HIF translocates to the nucleus, activating the transcription of target genes [13]. Although HIF1Alpha and HIF2Alpha share this regulatory mechanism, they play distinct roles. While HIF1Alpha is broadly expressed and mainly mediates the acute cellular response to hypoxia, HIF2Alpha is restricted to several cell types as the main transcriptional regulator of erythropoietin under hypoxic conditions [14].

According to this evidence, it may be hypothesized that both P and FGF23 might contribute to the development of anemia in renal dysfunction. However, these elements are closely interrelated. Thus, the aim of this work will be, on the one hand, to elucidate how the modulation of P load may impact the development of CKD-related anemia, particularly emphasizing the potential effect of dietary P restriction. On the other hand, the sustained effect of FGF23 blockade on renal anemia will be assessed, both in the context of renal dysfunction and also in the normal setting, examining the molecular mechanisms that may underlie this effect.

## 2. Results

### 2.1. The Effect of Phosphate Load on Parameters of Renal Function and Mineral Metabolism

In CKD rats, the administration of increasing dietary P content did not modify the circulating concentrations of calcium (Table 1). However, the levels of plasma P progressively increased as the consumption of P was higher, reaching significantly increased levels in the group of animals receiving 1.2% P (6.08 ±1.36 vs. 4.11 ± 0.73 mg/dL in the healthy controls, *p* < 0.05) (Table 1). The concentration of creatinine in normal rats was 0.46 ± 0.08 mg/dL. As expected, all the Nx rats exhibited significantly increased creatinine levels (1.18 ± 0.23, 1.26 ± 0.19, and 1.19 ± 0.22 mg/dL for Nx0.2P, Nx0.6P, and Nx1.2P, respectively, *p* < 0.001), with concentrations that were statistically equal regardless of P consumption (Table 1). However, the levels of FGF23 varied according to the amount of P contained in the diet. Thus, uremic animals with dietary P restriction (610 ± 46 pg/mL) showed statistically similar levels to those found in the control group (392 ± 74 pg/mL). However, FGF23 was significantly increased in Nx0.6P animals (687 ± 159 pg/mL, *p* < 0.05), and this augmentation was further marked in the Nx1.2P group (2302 ± 207 pg/mL, *p* < 0.0001) (Table 1).

On average, urine calcium (Ca) excretion in healthy rats was 2.60 ± 1.11 mg/24 h. No significant changes were observed among CKD groups, although urine Ca tended to be progressively diminished as dietary P increased (4.86 ± 1.79, 3.81 ± 1.18, and 2.47 ± 1.12 mg/24 h for Nx0.2P, Nx0.6P, and Nx1.2P rats, respectively) (Table 1). The urine levels of P clearly reflected the amount of this mineral contained in the diet. The excretion of P in normal rats was 14.88 ± 4.09 mg/24 h. The restriction of P in the diet was associated with a tendency to lower phosphaturia (5.92 ± 1.12 mg/24 h), although without reaching statistical significance. The administration of normal dietary P produced a phosphaturia slightly higher than that observed in controls (22.33 ± 4.56 mg/24 h). Only the Nx groups feeding 1.2% P exhibited significantly higher urine P (35.64 ± 8.34 mg/24 h), statistically higher than the levels found in all the other experimental groups (*p* < 0.001 vs. control, *p* < 0.0001 vs. Nx0.2P, and *p* < 0.01 vs. Nx0.6P) (Table 1).

### 2.2. Variations in the Phosphate Load and Their Effects on Hematological Parameters in CKD

Figure 1 shows the hematological status assessed in all the experimental groups. Overall, all CKD animals exhibited diminished levels of hematocrit (Figure 1A) in comparison with healthy rats (43.25 ± 0.96%). The hematocrit observed in the Nx0.2P and Nx0.6P rats was 37.00 ± 1.41 and 40.60 ± 0.89%, respectively. The group of Nx rats receiving the highest amount of P in the diet was markedly inferior to the other uremic groups (30.00 ± 3.63%). A similar trend was found when hemoglobin was assessed, with marked reductions in Nx animals (9.23 ± 0.69, 9.48 ± 0.96, and 7.63 ± 1.20 g/dL for Nx0.2P, Nx0.6P, and Nx1.2P, respectively) when compared with controls (12.10 ± 1.16 g/dL) (Figure 1B). Serum iron level in healthy animals was 133 ± 28 mg/dL. In uremic animals, progressive reductions in serum iron were observed with higher amounts of dietary P (123 ± 58, 97 ± 26, and 89 ± 8 mg/dL for Nx0.2P, Nx0.6P, and Nx1.2P, respectively), but without reaching statistical significance (Figure 1C). The absolute reticulocyte count was also assessed, finding significant reductions in all the Nx groups compared with controls. Indeed, the reticulocyte production in CKD animals was significantly reduced when compared with healthy rats (3.28 ± 0.48) and progressively decreased as dietary P content was higher (2.40 ± 0.50, 2.17 ± 0.36, 1.71 ± 0.38% for Nx0.2P, Nx0.6P, and Nx1.2P, respectively), with no statistical differences among them (Figure 1D). By contrast, all the groups of uremic animals had a trend to higher hepcidin levels when compared with healthy rats (1023 ± 480 pg/mL). In this regard, statistical differences were observed in the Nx0.6P and Nx1.2P groups (2183 ± 620 and 2427 ±703 pg/mL), but not in the animals receiving the lowest amount of P (2174 ± 541 pg/mL) (Figure 1E). We did not find any statistical difference in the circulating concentrations of erythropoietin among groups, although their levels were progressively lower as the dietary amount of P was higher (Figure 1F).

Morphological examination of peripheral blood and bone marrow smears (Figure 1G–J) demonstrated progressive erythrocyte morphological heterogeneity in response to increasing dietary P in Nx animals. Whereas control animals maintained normal erythrocyte morphology, characterized by a homogeneous population of normocytic, normochromic red blood cells, Nx animals developed progressively more pronounced morphological abnormalities that closely paralleled the increasing dietary phosphorus load. Hence, peripheral blood smear examination revealed that animals receiving 0.2% and 0.6% P diets exhibited morphological features consistent with CKD-associated anemia, characterized by a mild increase in erythrocyte morphological heterogeneity, including mild anisocytosis, anisochromia, and an increasing proportion of erythrocytes exhibiting a tendency toward microcytosis and hypochromia. These abnormalities became most pronounced in the Nx1.2P group, which exhibited a markedly more heterogeneous erythrocyte population with a greater proportion of microcytic and hypochromic erythrocytes, moderate anisocytosis, mild poikilocytosis, and occasional echinocytes, which are findings consistent with a more advanced anemic phenotype associated with the uremic state (Figure 1G). Reticulocyte staining demonstrated a progressive decline in circulating reticulocyte counts with increasing dietary P loading, reaching its lowest levels in the Nx1.2P group (Figure 1H). These findings closely paralleled the bone marrow examination, which demonstrated preserved overall cellularity and maintenance of multilineage hematopoiesis across all experimental groups. Nevertheless, a progressive reduction in the erythroid compartment became evident as dietary P load increased, particularly in animals receiving the highest P diet, consistent with the development of a progressively hyporegenerative erythropoietic response (Figure 1I). Prussian blue staining remained positive in all experimental groups, demonstrating preserved bone marrow iron stores. However, Nx animals exhibited a trend toward greater staining intensity than control animals, particularly those exposed to the highest dietary P load. This pattern is consistent with reticuloendothelial iron sequestration and functional iron restriction, rather than depletion of bone marrow iron stores (Figure 1J). Collectively, these morphological findings closely paralleled the quantitative hematological results and demonstrated a progressive deterioration of the erythropoietic phenotype in response to increasing dietary phosphorus loading. This process was characterized by progressively greater erythrocyte morphological heterogeneity, declining erythropoietic activity, and preservation of bone marrow iron stores in a setting consistent with functional iron restriction.

### 2.3. The Effect of Dietary Phosphorus Content on HIF Signaling in CKD Animals

The impact of the administration of different amounts of dietary P in CKD animals on HIF signaling was examined at different levels. At a circulating level, only the rats receiving 1.2% P (42 ± 12 pg/mL) had significantly higher levels of HIF1Alpha compared with both normal rats and Nx rats with P restriction (19 ± 7 pg/mL and 25 ± 4 pg/mL, respectively) (Figure 2A). Also, the expression of HIF1Alpha was evaluated at the renal level by Western blot. Uremic animals tended to exhibit lower expression of nuclear HIF1Alpha when compared with controls (1.00 ± 0.49), with this reduction being significantly inferior in the groups receiving the highest amounts of P (0.39 ± 0.27 and 0.29 ± 0.14 for Nx0.6P and Nx1.2P groups, respectively) (Figure 2B). However, the renal expression of HIF2Alpha was similar among all groups (Figure 2C), as was HIF1Alpha expression in bone marrow (Figure 2D). Yet, the expression of HIF2Alpha in bone marrow was found to be reduced in all CKD groups (0.82 ± 0.04, 0.78 ± 0.04, and 0.83 ± 0.06 for Nx0.2P, Nx0.6P, and Nx1.2P, respectively) in comparison with controls (1.00 ± 0.10) (Figure 2E).

### 2.4. Univariate and Multivariate Analyses

Univariate correlation analyses were performed to evaluate the existence of associations between CKD- and P metabolism-related parameters, and factors related to the hematologic status of the animals. Thus, we found that plasma *p* levels were significantly associated with hematocrit (r = −0.554, *p* <0.05), hemoglobin (r = −0.452, *p* < 0.05), serum iron (r = −0.609, *p* < 0.01), and plasma HIF1Alpha levels (r = 0.433, *p* < 0.05) (Table 2). On the other hand, plasma creatinine levels exhibited negative correlations with hemoglobin (r = −0.671, *p* < 0.001), serum iron (r = −0.444, *p* < 0.05), percentage of reticulocytes (r = −0.641, *p* < 0.01), renal (r = −0.715, *p* < 0.01), plasma HIF1Alpha (r = 0.522, *p* < 0.05), and bone marrow HIF2Alpha expression (r = −0.699, *p* < 0.001) (Table 2). When we analyzed the associations of FGF23 with hematological parameters, we found that it was strongly correlated with hematocrit (r = −0.860, *p* < 0.0001), hemoglobin (r = −0.705, *p* < 0.001), serum iron (r = −0.601, *p* < 0.01), and reticulocyte count (r = −0.656, *p* < 0.01). In addition, the levels of FGF23 were significantly associated with renal (−0.520, *p* < 0.05) and circulating (r = 0.684, *p* < 0.01) HIF1Alpha (Table 2).

In multivariate lineal models adjusted by P and creatinine, ln(FGF) was inverse and independently associated with hemoglobin (β = −0.602, 95%CI −1.009 to −0.195, *p* < 0.01), hematocrit (β = −0.609, 95%CI −1.187 to −0.030, *p* < 0.05), and bone marrow HIF1Alpha expression (β = 0.518, 95%CI 0.111 to 0.924, *p* < 0.05) (Table 3 and Figure 3).

### 2.5. The Administration of FGF23 Antibodies Rescues Anemia Phenotype in CKD

Given that worsening of CKD-related anemia is associated with high levels of P and FGF23 rising concomitantly in the setting of renal dysfunction, and that both elements were closely associated with hematocrit and hemoglobin, a different experiment was performed based on the administration of AntiFGF23 antibody to CKD animals receiving 1.2% P in the diet, while another group of uremic animals receiving a high P diet remained untreated. Thus, this model allows assessing the consequences of concomitantly having high P and low FGF23 in the CKD setting.

Biochemical analysis of blood and urine samples is depicted in Table 4. Plasma calcium levels did not change among the experimental groups, although the Nx1.2P group receiving AntiFGF23 had slightly, but not significantly, lower calcemia (7.27 ± 2.65 mg/dL) when compared with the rats that remained untreated (10.19 ± 3.38 mg/dL). As expected, Nx1.2P rats had markedly higher levels of plasma P (6.66 ± 1.29 mg/dL, *p* < 0.05) compared with controls (3.87 ± 0.72 mg/dL), which were also accompanied by elevated FGF23, and the administration of AntiFGF23 was associated with even more marked hyperphosphatemia (14.67 ± 3.41 mg/dL, *p* < 0.05 vs. other groups). Similarly, creatinine was elevated in Nx1.2P animals (1.10 ± 0.18 mg/dL, *p* < 0.05) when compared with healthy rats (0.63 ± 0.10 mg/dL), and it was further increased in those animals receiving AntiFGF23 (2.01 ± 0.19 mg/dL, *p* < 0.001 vs. other groups). With respect to urine parameters, no significant differences were observed between healthy and CKD rats (0.59 ± 0.28 vs. 1.58 ± 0.25 mg/24 h), although the administration of AntiFGF23 produced a marked hypercalciuria (19.30 ± 6.18 mg/24 h, *p* < 0.0001 vs. other groups). By contrast, a notable hyperphosphaturia (164.80 ± 44.72 mg/24 h vs. 43.95 ± 11.40 mg/24 h in the control group, *p* < 0.001) was observed in Nx1.2P rats that was markedly reduced in those animals receiving AntiFGF23 (4.17 ± 0.004 mg/24 h, *p* < 0.001 vs. Nx1.2P).

Regarding the hematological status, the existence of CKD was associated with the development of anemia, as evidenced by significant decreases in hematocrit (31.57 ± 5.32 vs. 44.50 ± 4.66% in the control group) and the reticulocyte count (1.68 ± 0.40 vs. 3.23 ± 0.57% in the controls) (Figure 4A and Figure 4B, respectively). In this scenario, the administration of AntiFGF23 prevented the decrease in both parameters (40.00 ± 4.58 and 3.73 ± 0.62% for hematocrit and reticulocytes, respectively), reaching levels similar to those observed in healthy animals. In addition, hepcidin was found to be increased in the Nx1.2P group (2907 ± 555 vs. 1672 ± 560 pg/mL in healthy animals), but not in the uremic rats treated with AntiFGF23 (2487 ± 211 pg/mL) (Figure 4C). However, there were no differences in plasma erythropoietin between normal and CKD rats (29.72 ± 29.56 and 33.88 ± 30.02 pg/mL, respectively), but it was markedly increased in the Nx1.2P+AntiFGF23 experimental group (387.50 ± 46.58 pg/mL) (Figure 4D).

Morphological examination of peripheral blood and bone marrow smears was consistent with the hematological findings obtained from the quantitative analyses. Both healthy and Nx1.2P animals exhibited features that were similar to those observed in the previous experiment. The administration of the AntiFGF23 antibody (Nx1.2P+AntiFGF23) was associated with partial restoration of the morphological abnormalities observed in untreated Nx1.2P animals. Peripheral blood smear examination demonstrated a more homogeneous erythrocyte population, with reduced anisocytosis and recovery of a predominantly normocytic and normochromic phenotype (Figure 4E). Consistent with these findings, reticulocyte staining demonstrated a significant increase in circulating reticulocytes compared with untreated Nx1.2P animals, reflecting recovery of erythropoietic activity (Figure 4F). Bone marrow examination revealed a qualitative expansion of the erythroid compartment, characterized by increased representation of erythroid precursors at different stages of maturation, consistent with enhanced erythropoiesis (Figure 4G). Prussian blue staining continued to demonstrate preserved bone marrow iron stores, indicating that restoration of erythropoiesis occurred in the presence of maintained marrow iron availability (Figure 4H). Overall, these morphological findings were fully consistent with the quantitative hematological data and provide morphological evidence that FGF23 blockade promotes recovery of erythropoiesis, thereby contributing to the improvement of the anemic phenotype associated with experimental chronic kidney disease.

HIF signaling was also examined in this setting. Uremic animals without (33.49 ± 3.59 pg/mL) and the group with AntiFGF23 (48.99 ± 14.25 pg/mL) had significantly higher levels of circulating HIF1Alpha when compared with healthy animals (19.26 ± 6.98 pg/mL) (Figure 5A). At the renal level, the administration of AntiFGF23 to CKD animals was associated with a significant and marked decrease in HIF1Alpha expression (0.08 ± 0.05) compared with untreated animals (0.58 ± 0.09) (Figure 5B). Also, in the kidney, both Nx groups showed similar expression of renal HIF2Alpha (1.13 ± 0.06 and 1.12 ± 0.04 for Nx1.2P and Nx1.2P+AntiFGF23, respectively) compared with normal rats (1.00 ± 0.07) (Figure 5C). In bone marrow, no changes in HIF1Alpha expression were associated either with the occurrence of CKD (0.51 ± 0.36 vs. 1.00 ± 0.46 in controls) or the concomitant administration of AntiFGF23 (0.45 ± 0.15) (Figure 5D). When HIF2Alpha was analyzed, it was found that the reduction in the expression level observed in the Nx1.2P group (0.77± 0.12 compared with 1.00 ± 0.10 in normal rats) was not modified after the administration of AntiFGF23 (0.76 ± 0.11) (Figure 5E).

### 2.6. The Effect of High and Low FGF23 Levels in Healthy Rats

Then, an additional experiment was conducted to define the effects of both high and low levels of FGF23 in the normal setting. Thus, two different groups of healthy rats were treated with high levels of FGF23 and AntiFGF23, respectively. Given the small sample size, the AntiFGF23 group was excluded from the inferential statistical testing; individual data points for this arm are shown descriptively in the corresponding figures. With respect to mineral metabolism-related parameters, no differences were observed in plasma calcium according to changes in FGF23 levels, although the rats receiving FGF23 showed a tendency to lower calcium concentrations (8.34 ± 2.24, 5.74 ± 3.03, and 7.92 ± 0.66 mg/dL for control, FGF23, and AntiFGF23 groups, respectively) (Table 5). The administration of FGF23 produced a significant decrease in plasma P (4.76 ± 0.46 vs. 6.30 ± 0.47 mg/dL in the control group, *p* < 0.01) (Table 5). As expected, and given that renal function was unaffected, plasma creatinine levels in the group with high (0.56 ± 0.06 mg/dL) FGF23 were statistically equal to those found in the control group (0.63 ± 0.08 mg/dL) (Table 5). Also, the exogenous administration of FGF23 was evidenced by a significant increase in FGF23 plasma levels (336 ± 63 vs. 248 ± 48 pg/mL in healthy rats, *p* < 0.05) (Table 5). When urine calcium was analyzed, rats receiving FGF23 were found to have slightly lower levels in comparison to controls (0.10 ± 0.07 vs. 0.20 ± 0.08 mg/24 h, respectively) (Table 5). By contrast, exogenous FGF23 produced a nonsignificant increase in phosphaturia (22.75 ± 11.75 vs. 20.79 ± 6.42 mg/24 h) (Table 5).

Regarding the hematological status, those rats receiving high doses of FGF23 exhibited a marked and significant decrease in the levels of serum iron when compared with controls (64.75 ± 17.29 vs. 130.00 ± 15.51 mg/dL) (Figure 6A). Also, FGF23-treated rats had a slight tendency to lower hematocrit (Figure 6B) and hemoglobin (Figure 6C). When the percentage of reticulocytes was evaluated, a significant reduction was observed in animals receiving FGF23 (1.56 ± 0.46 vs. 2.46 ± 0.26% in controls) (Figure 6D). Interestingly, hepcidin concentration was found to be increased in the animals treated with FGF23 (4.20 ± 0.86 vs. 2.88 ± 0.43 pg/mL) (Figure 6E). A significant increase in erythropoietin was associated with the administration of FGF23 (658 ± 260 vs. 213 ± 43 pg/mL) (Figure 6F).

The morphological assessment of peripheral blood and bone marrow smears demonstrated that the modulation of FGF23 was sufficient to alter erythropoietic activity in the absence of CKD. The control group displayed preserved hematologic morphology. The exogenous administration of FGF23 was associated with a significant reduction in circulating reticulocyte counts, accompanied by a reduction in the bone marrow erythroid compartment, consistent with decreased erythropoietic output. In contrast, the pharmacological blockade of FGF23 using a neutralizing antibody produced the opposite effect, resulting in a significant increase in circulating reticulocytes and a qualitative expansion of the erythroid compartment, with greater representation of erythroid precursors at different stages of maturation, consistent with enhanced erythropoiesis. Prussian blue staining remained positive in all experimental groups, without appreciable differences in either the distribution or intensity of bone marrow iron deposits, indicating that the observed changes in erythropoietic activity occurred in the preserved bone marrow iron stores. (Figure 6G–J).

The circulating concentration of HIF1Alpha as well as the expression of HIF1Alpha and HIF2Alpha in both kidney and bone marrow was also examined in this experiment. High concentrations of FGF23 did not modify HIF signaling under conditions of normal renal function (Figure 7).

## 3. Discussion

Anemia is a common complication of CKD associated with lower quality of life [15] and faster renal progression and mortality [16], among other undesirable outcomes. This study shows that the progressive increase in P load and the subsequent elevation in FGF23 lead to a worsening of anemia in experimental CKD. In this scenario, the blockade of FGF23 by administering AntiFGF23 antibodies completely abolished the signs of anemia, an effect that occurs in parallel with changes in renal HIF1Alpha signaling. However, under normal renal function, neither FGF23 administration nor AntiFGF23 treatment influenced HIF signaling, despite the marked effect of elevated FGF23 levels on iron metabolism in these healthy animals.

In this study, we used a preclinical model of CKD based on subtotal Nx together with the administration of different amounts of P to evaluate their impact on the development of anemia of renal origin. All uremic animals exhibited a similar degree of renal function based on the level of plasma creatinine. However, we observed the expected changes in phosphatemia, phosphaturia, and FGF23 according to the amount of P ingested. Furthermore, the administration of a deficient P diet prevented the development of hyperphosphatemia and hyperphosphaturia, as well as the elevation in FGF23, as we have previously shown [17]. When it comes to hematological parameters, although all the rats that developed CKD also had decreased hematocrit and hemoglobin, the administration of higher amounts of P produced an aggravation of this anemia, as evidenced by progressively lower hematocrit and hemoglobin values along with a further reduction in the number of reticulocytes. This finding reinforces the idea that FGF23 production is primarily driven by P balance rather than the decrease in renal dysfunction, rising as an adaptive response to counteract the early P retention [18]. Importantly, these results imply that, even with the same reduction in renal mass, the early dietary P restriction may blunt the elevation of FGF23 and attenuate its deleterious impact in the development of anemia, which supports the control of dietary P as an additional therapeutic strategy in its clinical management. The relationship between P and anemia found in our preclinical model of renal dysfunction is consistent with previous findings from the clinical setting. Thus, it has been proposed that in CKD high P correlates with a greater likelihood for anemia [8] and is independently associated with low hemoglobin levels [7,9]. Furthermore, the presence of hyperphosphatemia may result in hyporesponsiveness to therapy with erythropoiesis-stimulating agents [19,20]. A direct effect of the high levels of polyamines present in the serum of uremic patients seems to be at least partially responsible for this effect, given their inhibitory effect on the proliferation and maturation of erythroid precursor cells [21]. This finding is consistent with the pathological changes observed with the aid of specific staining techniques in smears obtained from peripheral blood and bone marrow. Taken together, these findings indicate that in the context of renal dysfunction, the P loading along with the subsequent elevation in FGF23 are relevant determinants for the development of anemia, and not merely the reduction in the glomerular filtration rate. In this regard, it should be remarked that hematocrit was slightly higher in the 0.6% P uremic group than in the group of CKD rats with P restriction (0.2% P), apparently contradicting a simple linear relationship between dietary P and improved erythropoiesis. Importantly, however, the lowest levels for both parameters were observed in the Nx1.2P group across the entire P gradient, indicating that excessive dietary P intake was associated with anemia worsening in our model. Our data regarding diet intake (Supplementary Figure S1) offers a plausible explanation for this deviation, given that cumulative food intake was lower in Nx0.2P rats compared to the Nx0.6P group, indicating a non-linear, threshold-like relationship between dietary P content and food intake. In addition, body weight (Supplementary Figure S2) was preserved in Nx0.2P relative to control, and serum iron was not reduced either, arguing against malnutrition or iron deficiency as explanations. Also, measured dietary P intake (Supplementary Figure S3) confirmed a dose-dependent gradient across groups, ruling out inadequate dietary compliance. Taken together, we interpret the relationship between dietary P and hematological status as non-linear rather than strictly linear: the most severe anemia occurred under the highest P intake, supporting the therapeutic rationale for P restriction in CKD, whereas the milder discrepancy observed at the lowest P intake most likely reflects a modest reduction in food intake rather than a failure on the proposed mechanism. In this sense, we acknowledge that nutritional parameters such as serum protein or albumin were not determined, which represents an important direction for future work.

Although hyperphosphatemia and high FGF23 are closely related, given that P is the major inducer of FGF23 production, different works have also gone in depth on the independent impact of FGF23 on erythropoiesis and the development of anemia. In the clinical setting, Bielesz et al. found that in CKD patients stages 1–5, cFGF23, comprising c-terminal fragments and the intact form of FGF23, is associated with low hemoglobin levels, more than the biologically active intact FGF23 [22]. The results reported by Nam, also in non-dialysis CKD patients, showed that those subjects with high cFGF23 levels had a higher likelihood of developing anemia, suggesting that this factor may be considered as a predictor of anemia in CKD [23]. In our work, the intact and biologically active form of FGF23 was also found to be associated with these parameters. Also, in dialysis, elevations in FGF23 were found to be associated with hyporesponsiveness to erythropoiesis-stimulating agents [24]. In mice, it has been evidenced that FGF23 impairs erythropoiesis, in an effect that is independent of changes in other factors like vitamin D, another primary modulator of FGF23 levels. In fact, erythroid precursors are susceptible to being affected by changes in FGF23 given that they express FGF23 receptors 1–4, as well as Klotho [25]. However, it cannot be ruled out that the deficiency in Klotho that characterizes CKD [26] may also be present in these precursor cells, also contributing to abnormal erythropoiesis regardless of changes in FGF23. The beneficial effect of the neutralization of FGF23 on erythropoiesis in the context of renal dysfunction has already been reported. Hence, Agoro et al. found that the administration of a specific blocking peptide to inhibit FGF23 signaling stimulated erythropoiesis and abolished anemia. In our experiments, FGF23 blockade was carried out using an AntiFGF23 antibody. This experimental model was validated by the expected changes in parameters of mineral metabolism, mainly exacerbated hyperphosphatemia and decreased phosphaturia. In addition, we observed increased calciuria and worsening of renal function, evidenced by elevated plasma creatinine concentrations. In this regard, it should be acknowledged that a limitation of this study concerns the interpretation of plasma FGF23 values in the AntiFGF23-treated groups. The ELISA kit used for FGF23 determination is a two-site sandwich assay that relies on two antibodies to distinguish N- and C-terminal epitopes of the intact hormone. The experimental AntiFGF23 administered precludes reliable determination of circulating FGF23 in treated animals. Hence, the absence of measurable FGF23 in these groups should not be interpreted as evidence of complete biological neutralization but as a methodological limitation inherent to the assay method. In terms of hematological parameters, the administration of AntiFGF23 resulted in normalization of hematocrit and reticulocytes, as well as attenuated elevation of hepcidin. All these effects were consistent with morphological changes found in peripheral blood and bone marrow. Remarkably, these effects were accompanied by inhibition of HIF1Alpha signaling at the renal level. Interestingly, in our experiments, we consistently observed a drastic decrease in renal HIF1Alpha expression in animals with renal dysfunction in comparison with healthy tissue, particularly reduced in those animals receiving high amounts of P. This observation was in line with findings that were previously reported in a histopathological study performed in biopsies from nephrectomized kidneys, showing that HIF1Alpha abundance is lower as renal function deteriorates, correlating with factors such as serum creatinine, estimated glomerular filtration rate, glomerular sclerosis, and tissue fibrosis [27]. While AntiFGF23 treatment improved erythropoiesis, it simultaneously produced marked hyperphosphatemia and worsened plasma creatinine, indicating that the beneficial effect on anemia came at the cost of aggravated mineral and renal impairment. This pattern was previously observed by Shalhoub et al., who showed that sustained FGF23 neutralization in a model of CKD produced improvement of secondary hyperparathyroidism but aggravated hyperphosphatemia and increased vascular calcification, ultimately resulting in increasing mortality [28]. These findings underscore that, despite its maladaptive role in anemia and cardiovascular complications in CKD, FGF23 still exerts an essential adaptive function as CKD progresses. Therefore, any therapeutic strategy aimed at FGF23 suppression must carefully balance this benefit against the risk of exacerbating P retention and CKD progression and should be evaluated along with other strategies capable of mitigating this trade-off.

The assessment of HIF signaling provides a mechanistic link between P, FGF23, and the anemia phenotype. In our experiments, the abundance of HIF1Alpha in the kidney was progressively reduced as P and FGF23 levels were higher. In addition, the sustained blockade of FGF23 was associated with a marked change in renal HIF1Alpha while normalizing hematocrit and the percentage of reticulocytes. Iron deficiency determines an increase in FGF23 transcription *via* activation of HIF1Alpha [29,30]. Our results support the notion of a bidirectional loop, in which FGF23 suppression is accompanied by HIF1Alpha activation and reversion of anemia phenotype, even though plasma HIF1Alpha levels remain high. In this regard, the tissue- and isoform-specific pattern of these changes suggests that FGF23-induced regulation of HIF may differ across tissues, given that renal HIF2Alpha and bone marrow HIF1Alpha remained relatively unaffected while in bone marrow HIF2Alpha was reduced in all CKD groups independently of P intake. Importantly, the reduction in renal HIF1Alpha should be interpreted with caution. Canonically, renal erythropoietin production is driven by HIF2Alpha [31]. Given that HIF2Alpha expression remained unchanged in our model, the decrease in HIF1Alpha may be a secondary consequence of attenuation of hypoxia rather than a direct driver of the erythropoietic response. Alternative or complementary mechanisms, regardless of HIF signaling, may contribute to explaining the rise in erythropoietin, such as FGF23-mediated modulation of hepcidin [32], which enhances iron availability, or effects of FGF23 on erythroid progenitors, as has been recently published [33].

An additional finding of this study is that the detrimental effect of high FGF23 at the hematological level is not limited to the presence of renal dysfunction. In the normal setting, the administration of FGF23 produced a marked decrease in serum iron that was accompanied by a reduction in the number of immature red blood cells. This effect may be mediated by a direct stimulation of hepcidin [32], which is a potent inhibitor of iron absorption and trafficking from the reticulo-endothelial system into plasma [34], an effect that was observed without any involvement of HIF signaling. However, it is plausible to hypothesize that a longer-term administration of FGF23 in the context of normal renal function would eventually lead to the onset of anemia given the tendency to lower hematocrit and hemoglobin observed in our experimental setting. On the other hand, another interesting finding of this study was the marked increase in erythropoietin production associated with FGF23 blockade in CKD animals. Erythropoietin has been shown not only to promote FGF23 transcription but also the proteolytic cleavage, which results in a net reduction in the intact to c-terminal FGF23 ratio [35]. In the renal context, the stimulatory effect on erythropoietin production of FGF23 blockade might represent a novel and beneficial effect of lowering FGF23 therapeutic strategies regarding the hematological status.

Finally, our study is not exempt from limitations that should be acknowledged. First, our experiments are based on the use of subtotal Nx. Although this experimental model closely resembles the main features of CKD, it does not completely recapitulate the etiological heterogeneity and the chronicity of human CKD, which may limit the full and immediate extrapolation of the results into clinical practice. Group sizes were determined based on prior experience with this animal model rather than a formal a priori power calculation. While the CKD model closely resembled previous findings in the field, future studies would benefit from power calculations based on pilot data or previously published effect sizes to more accurately determine the optimal sample size. In this regard, the small size of some of the experimental groups due to postsurgical deaths may limit the statistical power, although significant differences were still found among treatments. In particular, we must acknowledge that the AntiFGF23 group with normal renal function included a small number of animals due to the limited availability of the experimental antibody used. Because this restricts the statistical power of comparisons involving this group, future studies with larger sample sizes would help confirm and extend these findings. Finally, this study was performed in male Wistar rats and, therefore, gender-related differential impact on CKD progression and/or hematological alterations cannot be discarded.

In sum, the findings reported here show that the modulation of P load has an impact on the severity of anemia in the setting of chronic renal dysfunction, remarking the beneficial effect of limiting dietary P. In this situation, the management of FGF23 levels is associated with amelioration of anemia and restoration of hematological parameters, an effect that is accompanied by changes in renal HIF1Alpha expression. In addition, we also show that the presence of high FGF23 in the context of normal renal function leads to abnormal iron metabolism with no alterations in HIF signaling. Taken together, these results support the notion of abnormally high FGF23 as a factor whose control may be beneficial in terms of onset and progression of anemia of renal origin. However, it is worth noting that fully neutralizing FGF23 in this scenario also leads to exacerbated hyperphosphatemia and poorer renal function. In this sense, any therapeutic strategy oriented towards blocking FGF23 needs to be carefully monitored to avoid undesirable outcomes. This is an aspect that should be specifically addressed in further studies exploring FGF23-lowering therapies as a strategy for the clinical management of CKD-related anemia.

## 4. Material and Methods

### 4.1. Animal Care

Animal care and experimental procedures were approved by the Research and Ethics Committee of the University of Cordoba and by Junta de Andalucia in accordance with Directive 2010/63/EU of the European Parliament, with institutional guidelines for the care and use of laboratory animals and the Declaration of Helsinki. Animals received humane care in compliance with the Principles of Laboratory Animal Care from the National Society for Medical Research.

Healthy male Wistar rats (Charles River Laboratories, Wilmington, MA, USA), 9–10 weeks old and weighing 250–300 g, were individually housed using a 12 h/12 h light/dark cycle. Animals were given ad libitum access to a standard diet (Altromin, Lage, Germany). The experimental groups included an average of 5 animals, and the study included a total of 52 rats. In all experiments, a group of healthy or untreated rats was used as a control. Group sizes were based on prior experience with this animal model, following the 3Rs principle (Replacement, Reduction, and Refinement) to minimize animal use. It was confirmed that all rats were visibly healthy before the study; no exclusion criteria were adopted for the exclusion of animals during the experiments, and no specific criteria were set for data points during the analyses. Animals were randomly allocated into the different experimental groups. Potential confounders, such as the order of treatments and measurements, or animal/cage location, were not controlled. The first and second authors of the study were aware of the group allocation at different stages of the experiment and during the subsequent data analysis.

#### 4.1.1. Experimental Model 1: The Effect of Phosphate Load in CKD

Renal failure was induced by subtotal (5/6) Nx, a two-step procedure. In the first step, animals were anesthetized using sevoflurane. Briefly, a 5 to 8 mm incision was made on the left mediolateral surface of the abdomen. The left kidney was exposed, and the two poles (two-thirds of the renal mass) were ablated. After one week, animals underwent right Nx under anesthesia. Control rats underwent a sham operation. To test the contribution of P load on the development of anemia, uremic rats were distributed into the experimental groups receiving diets containing the following amounts of P: 0.2% (Nx0.2P), 0.6% (Nx0.6P), and 1.2% (Nx1.2P) for 28 days. Animals were housed in metabolic cages to collect 24 h urine samples before sacrifice.

#### 4.1.2. Experimental Model 2: The Effect of the Administration of AntiFGF23 Antibody and High Phosphate in CKD

To assess the effect of suppressing FGF23 in the development of CKD-related anemia, a group of Nx rats fed a diet containing 1.2% P was treated with an AntiFGF23 antibody at a dose of 1 mg/kg sc on alternate days for 28 days.

#### 4.1.3. Experimental Model 3: The Effect of Low and High FGF23 Concentrations in Rats with Normal Renal Function

In addition, a pilot experiment was carried out to determine the effect of both the presence and absence of FGF23 on hematological parameters. Hence, a group of normal Wistar rats fed a diet with normal P content (0.6%) was treated with recombinant FGF23 (kindly donated by Amgen, Thousand Oaks, CA, USA) at a dose of 15 µg/day. Briefly, rats were anesthetized using sevofluorane, and an Alzet^®^ mini-osmotic pump (Charles River Laboratories, Barcelona, Spain) was implanted subcutaneously between the shoulders and loaded with either recombinant FGF23 or vehicle. Recombinant FGF23 was infused over 28 days as previously described [36]. On the other hand, another group of healthy animals was treated with AntiFGF23 antibody at a dose of 1 mg/kg sc on alternate days as in experimental model 2.

### 4.2. Parameters of Renal Function and Mineral Metabolism

After 28 days, animals were sacrificed and exsanguinated under general anesthesia with sevoflurane. Blood was collected in heparinized syringes, and plasma was separated by centrifugation and stored at −80 °C until subsequent processing. Serum and urine total calcium, phosphorus, and creatinine were determined by spectrophotometry (Biosystems, Barcelona, Spain). An enzyme-linked immunosorbent assay (ELISA) kit was used for the measurement of intact FGF23 (Kainos Laboratories, Tokyo, Japan).

### 4.3. Hematological Parameters

Whole blood samples were collected to determine both hematocrit and hemoglobin. Specifically, a microcentrifuge was used for assessment of hematocrit, while hemoglobin levels were analyzed by spectrophotometry (Thermo Fisher Scientific, Waltham, MA, USA). Serum iron was quantified with the aid of spectrophotometric techniques (Biosystems). The concentration of the factors related to iron metabolism, hepcidin, erythropoietin, and HIF1alpha, was assessed with ELISA kits from Cusabio (Wuhan, China) and Antibodies Online (Limmerick, PA, USA).

The reticulocyte count was performed from peripheral blood smears stained with Brilliant Cresyl Blue (Thermo Fisher Scientific). Cells were observed under light microscopy, identifying reticulocytes by the presence of reticulo-filamentous granules in the cytoplasm. For morphological assessment, peripheral blood and bone marrow smears stained with May–Grünwald–Giemsa (Sigma-Aldrich, St. Louis, MO, USA) were prepared to analyze the different stages of erythroid maturation. Additionally, bone marrow smears were stained with Prussian blue (Sigma-Aldrich) to qualitatively assess the iron content at the bone marrow level.

### 4.4. Protein Isolation and Western Blot Analysis

Protein extraction was carried out as previously described [37]. Briefly, kidney samples were homogenized at 4 °C in buffer A (pH 7.9) containing 10 mM HEPES, 10 mM KCl, 0.1 mM EDTA, 0.1 mM EGTA, 1 mM DTT, 0.5 PMSF, 15 µL/mL Protease Inhibitor Cocktail, and 0.5% Igepal Ca-630 (all reagents were purchased from Sigma), incubated for 15 min on ice, and centrifuged at 13,000 rpm for 3 min at 4 °C. The supernatant was removed, and the pellet was resuspended with cold buffer B (pH 7.9) prepared with 20 mM HEPES, 0.4 M NaCl, 1 mM EDTA, 1 mM EGTA, 1 mM DTT, 0.5 mM PMSF, and 15 µL/mL Protease Inhibitor Cocktail. After 15 min of incubation on ice, the extracts were centrifuged at 13,000 rpm for 5 min at 4 °C, and the supernatant containing the nuclear protein fraction was stored at −80 °C until analysis. Protein concentration was determined by Bradford assay (Bio-Rad Laboratories, Hercules, CA, USA). To determine specific protein contents, 75 µg of nuclear protein extracts were electrophoresed on a polyacrylamide gel (Bio-Rad) and electrophoretically transferred (Transfer Systems, Bio-Rad) from the gels onto nitrocellulose membranes (Bio-Rad). The following steps were performed with gentle shaking. Membranes were incubated in TTBS solution (20 mM Tris-HCl, 0.2% Tween 20, 150 mM NaCl) with 5% non-fat dry milk (all reagents purchased from Sigma) at room temperature for 1 h to avoid non-specific binding. Membranes were then washed with TTBS buffer and incubated overnight at 4 °C with a 1:500 dilution of a rabbit anti-HIF1Alpha antibody (Abcam, Cambridge, UK). Afterward, membranes were again washed with TTBS buffer and immunolabeled with an appropriate peroxidase-conjugated secondary antibody (Santa Cruz Biotechnologies, Santa Cruz, CA, USA). Finally, they were developed using Clarity Western ECL Blotting Substrate (Bio-Rad). Protein levels were quantified using ImageJ software1.53e (National Institutes of Health, Bethesda, MD, USA). Quantitative analysis was normalized to total protein content as assessed by Ponceau S staining.

### 4.5. Immunofluorescence

The renal expression of HIF2Alpha was evaluated by immunofluorescence. Kidney tissue was fixed in paraformaldehyde, paraffin-embedded, and sectioned (3–4 µm) for immunofluorescence. After deparaffinization, hydration, antigen retrieval in pH 6.0 citrate buffer, and permeabilization, sections were incubated overnight at 4 °C with an anti-HIF2Alpha antibody (ThermoFisher Scientific, Waltham, MA, USA) at 1:100 dilution, followed by Alexa Fluor 647-conjugated secondary antibody. Nuclei were counterstained with DAPI. Negative controls were performed by omitting the primary antibody. Images were acquired with the aid of a Thunder fluorescence microscope (Leica, Wetzlar, Germany) and analyzed using AIVIA software 13.1.0 (Bellevue, WA, USA).

### 4.6. Immunohistochemistry

Immunohistochemical techniques were used for HIF1Alpha and HIF2Alpha in bone tissue. Briefly, left femora were fixed in 4% formalin and embedded in paraffin after decalcification with ethylenediaminetetraacetic acid (EDTA) (10%; pH 7.4) for 30 days at 4 °C in agitation. Next, 4 µm sections were deparaffinized in xylene and rehydrated in ethanol. Immunostaining was performed using the ImmPRESS HRP Universal and Polymer kit (Vector Laboratories, Newark, CA, USA). Sections were then incubated overnight at 4 °C with rabbit anti-HIF1Alpha (Servicebio, Wuhan, China) and mouse anti-HIF2Alpha (ThermoFisher Scientific) antibodies at 1:100 and 1:200 dilutions, respectively. Sections were counterstained with haematoxylin for 30 s (PanReac AppliChem ITV Reagents, Darmstadt, Germany) and dehydrated. Staining with only polymer without primary antibody was used as a negative control to avoid nonspecific signals. Images were analyzed using QuPath software 0.7.0 [38].

### 4.7. Statistical Analysis

Values are expressed as the mean ± standard deviation. Differences between group means were assessed using Student’s *t*-test for two groups or ANOVA followed by Duncan’s post hoc test for three or more groups, or their corresponding non-parametric tests. A linear regression or curve fitting test was performed to assess the correlations between two variables. Multiple regression analysis was used to analyze the effect of multiple independent variables on FGF23 levels. A *p*-value inferior to 0.05 was considered to represent a statistically significant difference.

## Figures and Tables

**Figure 1:**
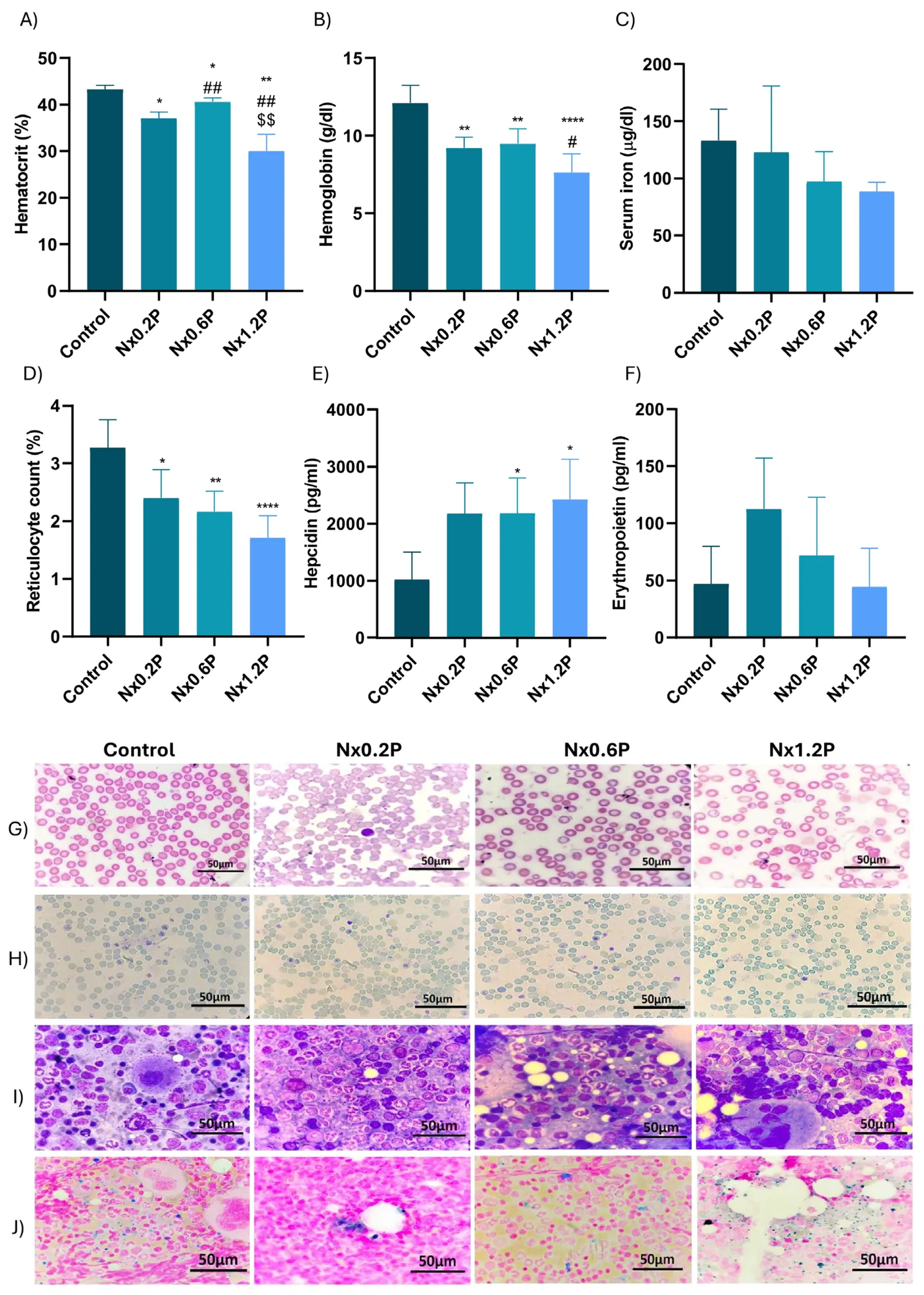
The effect of the consumption of increasing concentrations of phosphorus on hematological parameters in rats with chronic kidney disease. Hematocrit, hemoglobin, and reticulocyte count were significantly reduced in all the groups of uremic rats (**A**,**B**,**D**), while serum iron tended to be lower, particularly in the groups receiving the highest concentrations of phosphorus (**C**). Hepcidin concentrations were markedly and significantly elevated in the groups feeding 0.6% phosphorus or above (**E**). The plasma levels of erythropoietin were progressively diminished when dietary phosphorus was more elevated, although the differences were not statistically significant (**F**). Specific staining for red blood cells and reticulocytes in peripheral blood and for cells and iron reserves in bone marrow smears showed changes that were consistent with the alterations observed in the abovementioned factors (**G**–**J**). Data are expressed as mean ± SD. Initial/final n per group: control (n = 4/4), Nx0.2P (n = 6/4), Nx0.6P (n = 6/6), and Nx1.2P (n = 8/7). Animal losses were due to postsurgical complications following nephrectomy. Nx, nephrectomy; P, phosphorus. * *p* < 0.05 vs. control; ** *p* < 0.01 vs. control; **** *p* < 0.0001 vs. control; ^#^ *p* < 0.05 vs. Nx0.2P; ^##^ *p* < 0.01 vs. Nx0.2P; ^$$^ *p* < 0.01 vs. Nx0.6P. Magnification: 100×. Scale bars: 50 µm.

**Figure 2:**
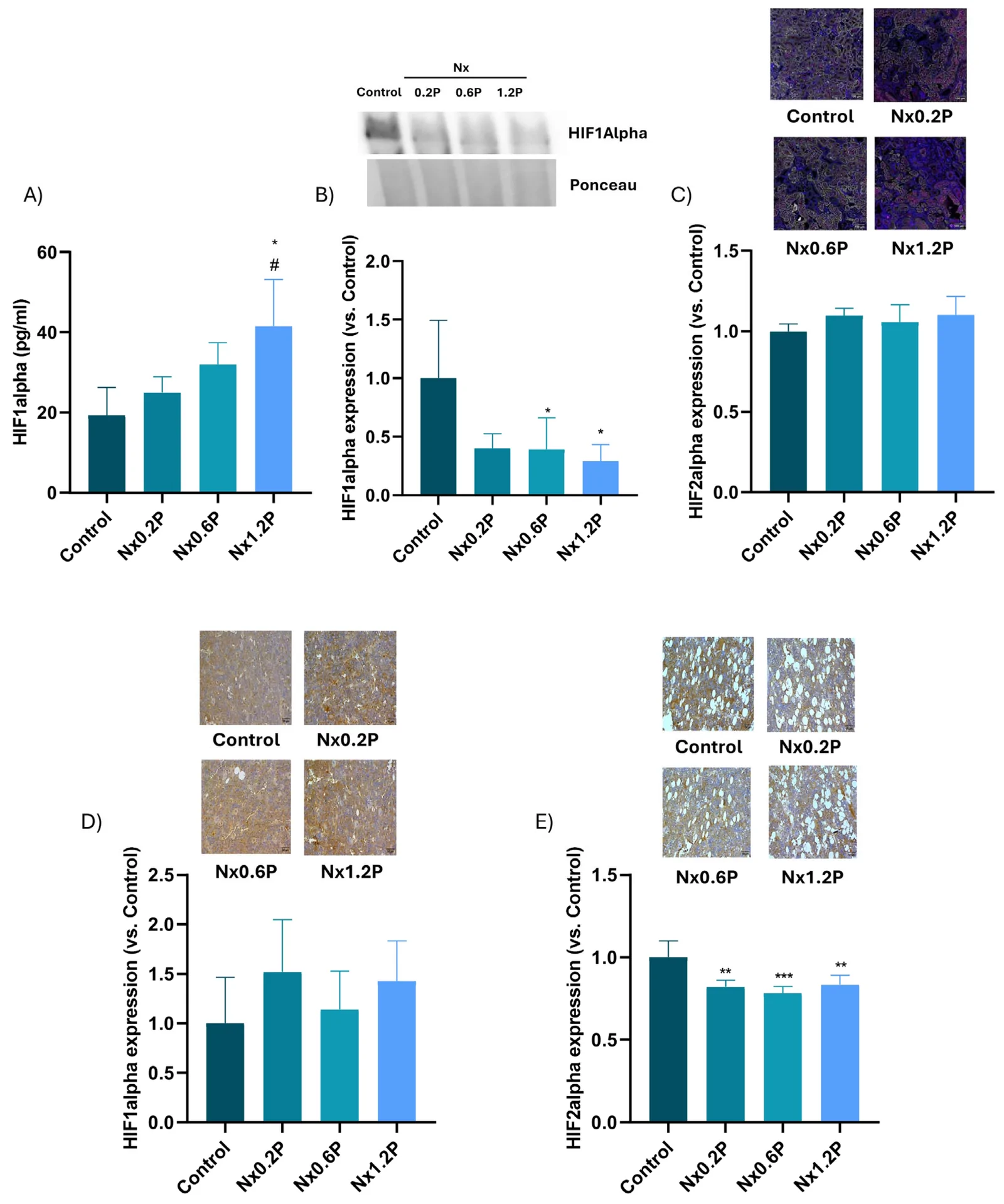
Hypoxia-inducible factor (HIF) signaling at different levels in animals with renal dysfunction receiving increasing amounts of phosphorus. In plasma, HIF1Alpha concentration tended to rise in uremic animals, although only rats receiving the highest amount of phosphorus exhibited significantly increased levels (**A**). At the kidney level, the presence of renal dysfunction was overall associated with lower renal HIF1Alpha expression, with marked differences in the groups fed 0.6% phosphorus or more (**B**). However, no differences were detected in renal HIF2Alpha and in bone marrow HIF1Alpha expression (**C**,**D**), neither between healthy and uremic rats nor among different phosphorus consumption. Also, in bone marrow, the expression of HIF2Alpha was significantly reduced in all groups of nephrectomized rats (**E**). Data are expressed as mean ± SD. Initial/final n per group: control (n = 4/4), Nx0.2P (n = 6/4), Nx0.6P (n = 6/6), and Nx1.2P (n = 8/7). Animal losses were due to postsurgical complications following nephrectomy. Nx, nephrectomy; P, phosphorus; HIF, hypoxia-inducible factor. * *p* < 0.05 vs. control; ** *p* < 0.01 vs. control; *** *p* < 0.001 vs. control; ^#^ *p* < 0.05 vs. Nx0.2P. Magnification: 40× (**C**), 20× (**D**,**E**). Scale bar: 100 µm (**C**), 50 µm (**D**,**E**).

**Figure 3:**
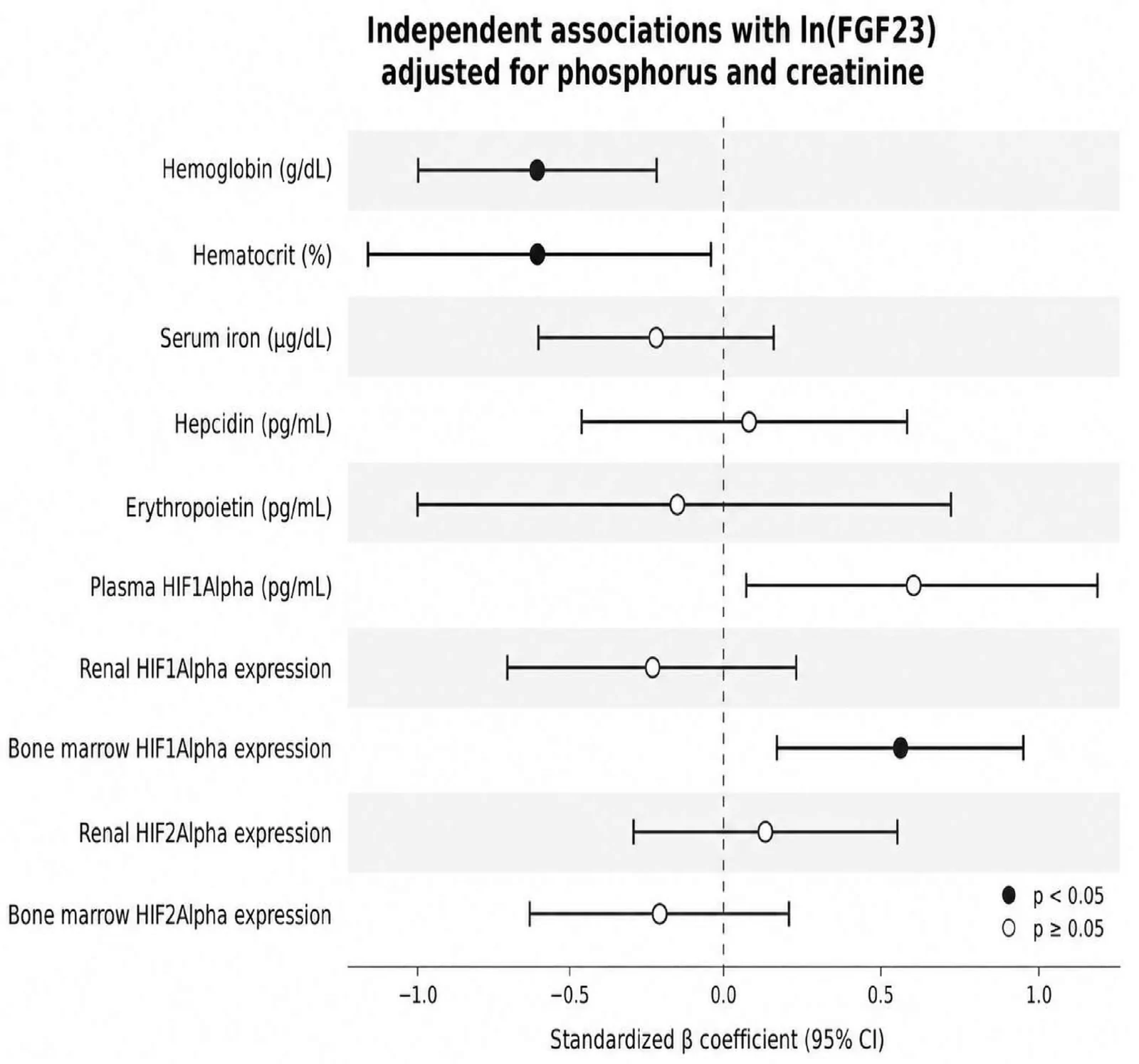
Forest plot of the multivariate analysis of the association between intact FGF23 and independent variables of interest, adjusted by plasma phosphorus and creatinine. Black points indicate a *p*-value inferior to 0.05, and white points refer to a *p*-value equal to or higher than 0.05. The most relevant independent association was found between intact FGF23 and hemoglobin and hematocrit.

**Figure 4:**
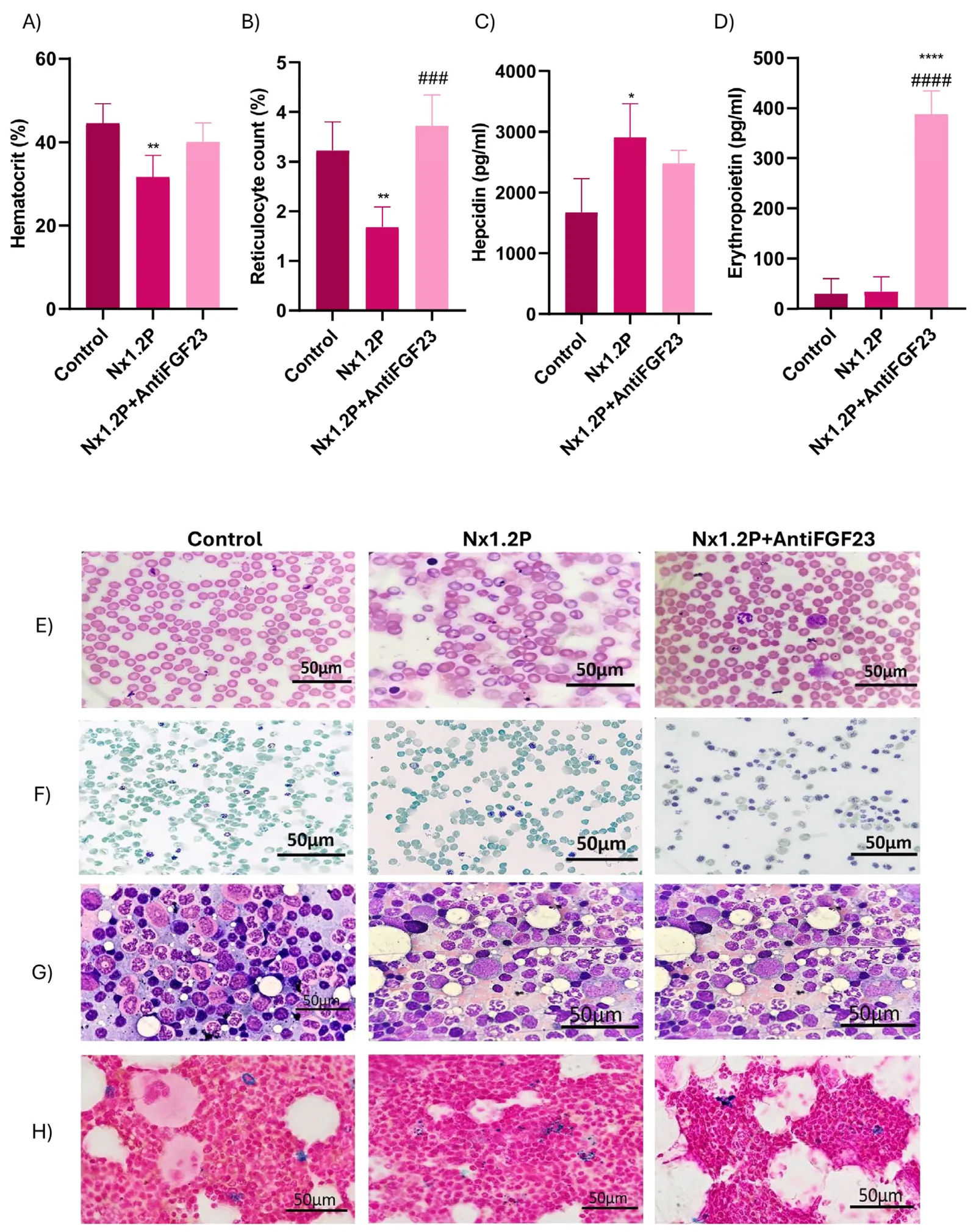
The effect on hematological status of the administration of AntiFGF23 to uremic rats receiving high amounts of dietary phosphorus. The presence of renal dysfunction along with the administration of high phosphorus produced a significant reduction in the hematocrit and the reticulocyte count, while the administration of AntiFGF23 under these conditions prevented the decrease in both parameters (**A**,**B**). Also, the Nx1.2P group had significantly elevated hepcidin levels that were not observed with the administration of AntiFGF23 (**C**). The administration of AntiFGF23 in uremic rats receiving high phosphorus was associated with a marked elevation in erythropoietin in plasma (**D**). Morphological assessment of peripheral blood and bone marrow revealed that the administration of AntiFGF23 promoted recovery of erythropoiesis, rescuing the anemia phenotype (**E**–**H**). Data are expressed as mean ± SD. Initial/final n per group: control (n = 5/5), Nx1.2P (n = 5/4), and Nx1.2P+AntiFGF23 (n = 6/4). Animal losses were due to postsurgical complications following nephrectomy. Nx, nephrectomy; P, phosphorus. * *p* < 0.05 vs. control; ** *p* < 0.01 vs. control; **** *p* < 0.0001 vs. control; ^###^ *p* < 0.001 vs. Nx1.2P; ^####^ *p* < 0.0001 vs. Nx1.2P. Magnification: 100×. Scale bars: 50 µm.

**Figure 5:**
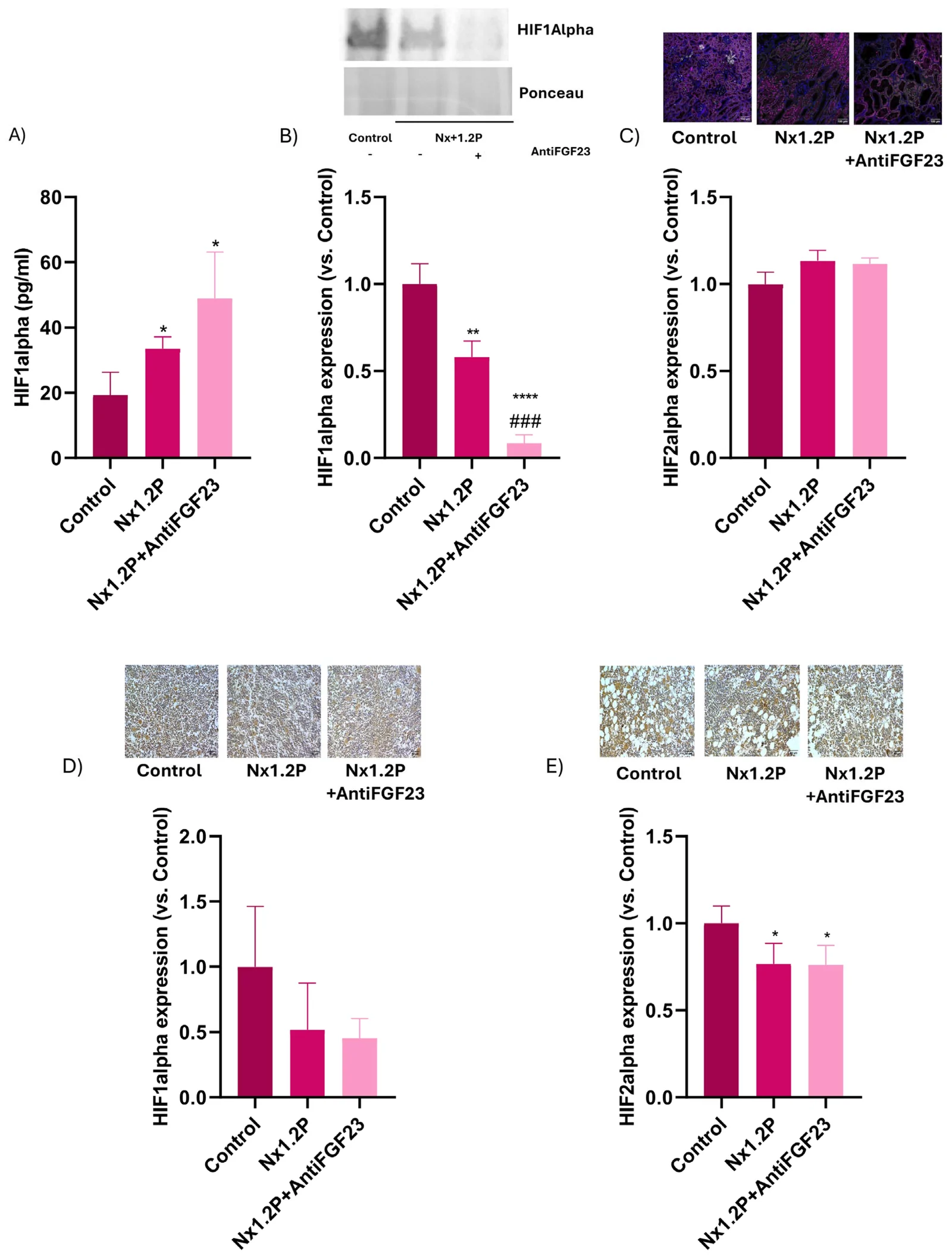
The effect on HIF signaling of the administration of AntiFGF23 to uremic rats receiving high amounts of dietary phosphorus. The circulating concentration of HIF1Alpha was significantly increased in the Nx1.2P group, and it was not modified by the administration of FGF23 antibodies (**A**). In the kidney, those animals treated with AntiFGF23 had a markedly reduced expression of HIF1Alpha (**B**), but it had no effect on HIF2Alpha expression (**C**). In bone marrow, the expression of both HIF1Alpha and HIF2Alpha showed a similar pattern, with a tendency to lower expression in both experimental groups with renal dysfunction (**D**,**E**). Data are expressed as mean ± SD. Initial/final n per group: control (n = 5/5), Nx1.2P (n = 5/4), and Nx1.2P+AntiFGF23 (n = 6/4). Animal losses were due to postsurgical complications following nephrectomy. Nx, nephrectomy; P, phosphorus. * *p* < 0.05 vs. control; ** *p* < 0.01 vs. control; **** *p* < 0.0001 vs. Control; ^###^ *p* < 0.001 vs. Nx1.2P. Magnification: 40× (**C**), 20× (**D**,**E**). Scale bar: 100 µm (**C**), 50 µm (**D**,**E**).

**Figure 6:**
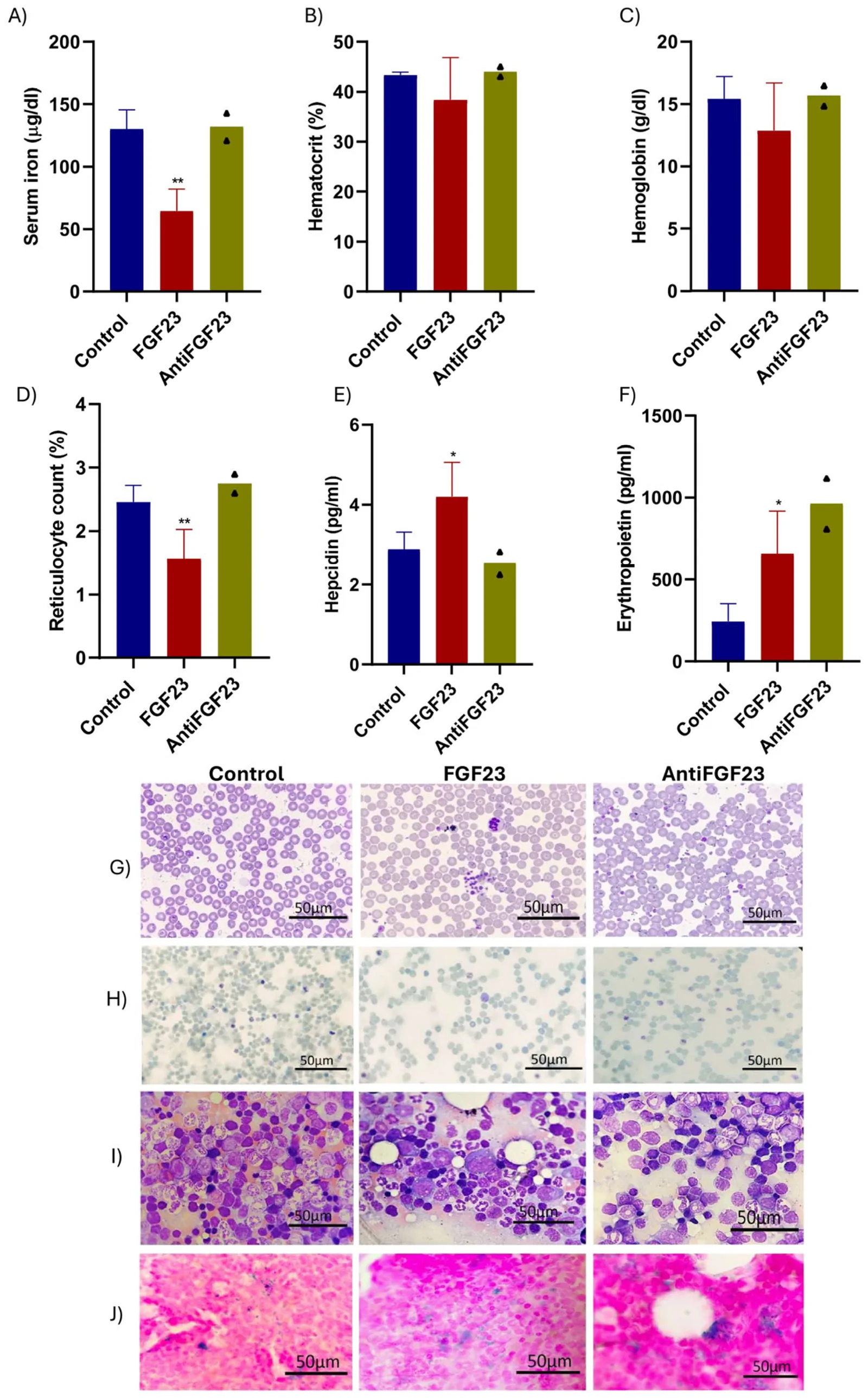
Modulation of the hematological status of healthy rats with high and low levels of FGF23. The administration of high concentrations of FGF23 was associated with a significant decrease in serum iron (**A**) and reticulocyte count (**D**) and a slight, but not significant, reduction in both hematocrit (**B**) and hemoglobin (**C**). In addition, high levels of FGF23 produce a marked increase in hepcidin (**E**) and erythropoietin (**F**) production. The administration of FGF23 produced a reduction in the number of reticulocytes accompanied by consistent changes in erythroid precursors in bone marrow. (**G**–**J**). Data are expressed as mean ± SD. Individual data points in AntiFGF23 group are shown as triangles (▲). Initial/final n per group: control (n = 5/5), FGF23 (n = 5/5), and AntiFGF23 (n = 2/2). Given the small sample size in the AntiFGF23 group, data involving this arm are only descriptive. * *p* < 0.05 vs. control; ** *p* < 0.01 vs. control. Magnification: 100×. Scale bars: 50 µm.

**Figure 7:**
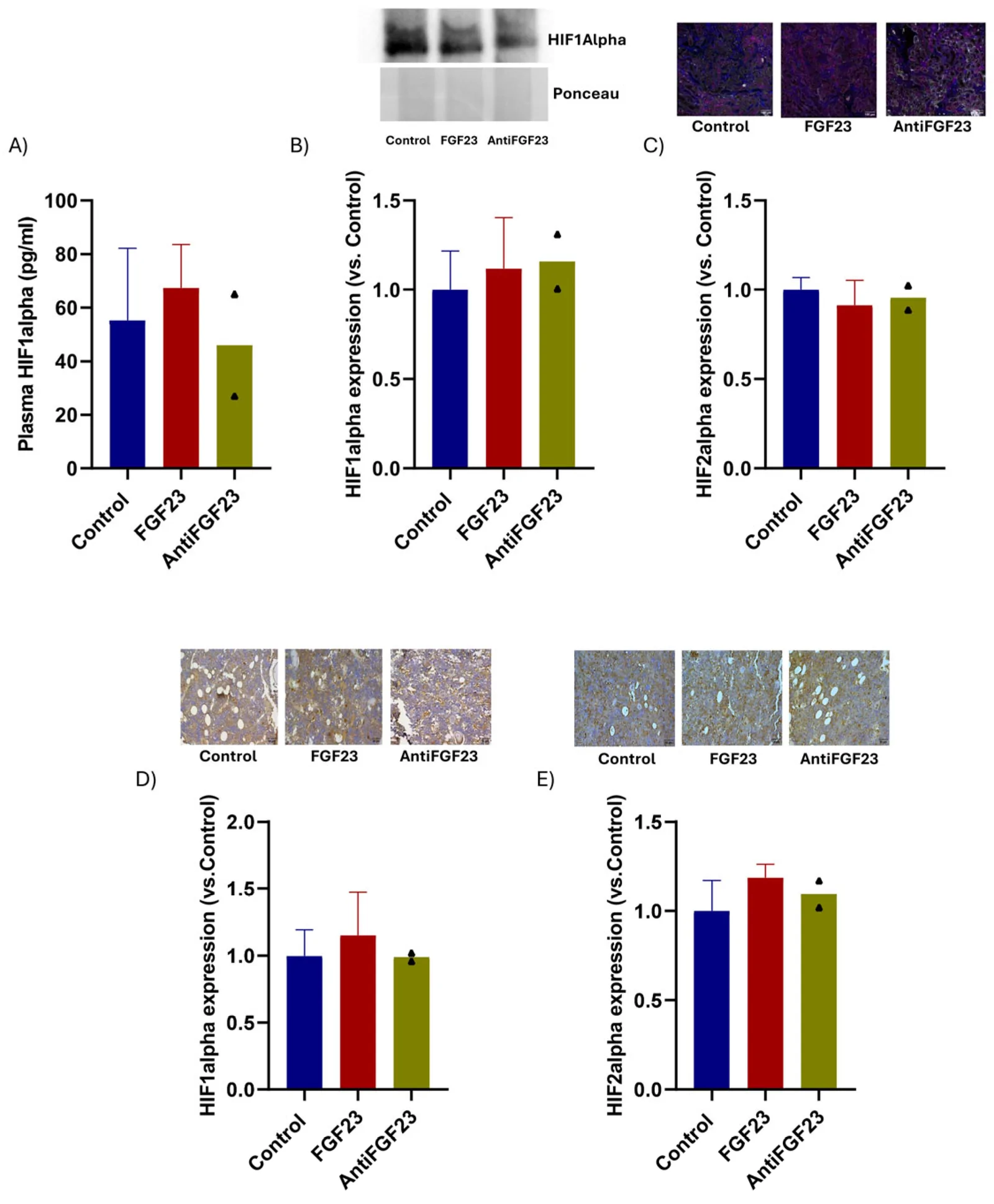
HIF signaling in rats with normal renal function treated either with high concentrations of FGF23 or AntiFGF23 antibodies. High concentrations of FGF23 did not produce any change in HIF signaling, determined according to circulating HIF1Alpha concentration (**A**), and renal and bone marrow expression of HIF1Alpha (**B**,**C**) and HIF2Alpha (**D**,**E**). Data are expressed as mean ± SD. Individual data points in AntiFGF23 group are shown as triangles (▲). Initial/final n per group: control (n = 5/5), FGF23 (n = 5/5), and AntiFGF23 (n = 2/2). Given the small sample size in the AntiFGF23 group, data involving this arm are only descriptive. Magnification: 40× (**C**), 20× (**D**,**E**). Scale bar: 100 µm (**C**), 50 µm (**D**,**E**).

**Table 1:** Parameters of mineral metabolism and renal function in normal rats and in animals with chronic kidney disease receiving different dietary phosphorus loads. Data are expressed as mean ± SD. Initial/final n per group: control (n = 4/4), Nx0.2P (n = 6/4), Nx0.6P (n = 6/6), and Nx1.2P (n = 8/7). Animal losses were due to postsurgical complications following nephrectomy. Nx: nephrectomy; FGF23: intact fibroblast growth factor 23. *, significantly different vs. control; ^#^, significantly different vs. Nx0.2P; ^$^, significantly different vs. Nx0.6P. Symbols may appear in combination when a value differs significantly from more than one reference group.

	Control	Nx0.2P	Nx0.6P	Nx1.2P
Plasma calcium (mg/dL)	10.49 ± 1.89	11.05 ± 1.85	11.16 ± 1.81	11.05 ± 2.04
Plasma phosphorus (mg/dL)	4.11 ± 0.73	3.61 ± 0.97	4.99 ± 0.64	6.08 ± 1.36 *^#^
Plasma creatinine (mg/dL)	0.46 ± 0.08	1.18 ± 0.23 *	1.26 ± 0.19 *	1.19 ± 0.22 *
Plasma FGF23 (pg/mL)	392 ± 74	610 ± 46	687 ± 159 *	2302 ± 207 *^#$^
Urine calcium (mg/24 h)	2.60 ± 1.11	4.86 ± 1.79	3.81 ± 1.18	2.47 ± 1.12
Urine phosphorus (mg/24 h)	14.88 ± 4.09	5.92 ± 1.12	22.33 ± 4.56 *	35.64 ± 8.34 *^#$^

**Table 2:** Correlation analyses of hematological parameters and phosphorus, creatinine, FGF23, and renal HIF1Alpha expression in pooled data collected from healthy rats and animals with chronic kidney disease receiving different dietary phosphorus loads. FGF23, fibroblast growth factor 23; HIF, hypoxia-inducible factor.

Reference Variable	Parameter	r Value (95% CI)	*p*-Value
Plasma phosphorus	Hematocrit (%)	−0.554 (−0.805 to −0.133)	0.0139
	Hemoglobin (g/dL)	−0.452 (−0.739 to −0.025)	0.0399
	Serum iron (mg/dL)	−0.609 (−0.828 to −0.228)	0.0034
	Plasma HIF1Alpha (pg/mL)	0.433 (0.002 to 0.729)	0.0498
Plasma creatinine	Hemoglobin (g/dL)	−0.671 (−0.855 to −0.337)	0.0009
	Serum iron (mg/dL)	−0.444 (−0.735 to −0.015)	0.0438
	Reticulocyte count (%)	−0.641 (−0.840 to −0.290)	0.0017
	Renal HIF1Alpha expression	−0.715 (−0.890 to −0.358)	0.0012
	Plasma HIF1Alpha (pg/mL)	0.522 (0.116 to 0.778)	0.0152
	Bone marrow HIF2Alpha expression	−0.699 (−0.869 to −0.382)	0.0004
FGF23	Hematocrit (%)	−0.860 (−0.945 to −0.665)	<0.0001
	Hemoglobin (g/dL)	−0.705 (−0.871 to −0.392)	0.0004
	Serum iron (mg/dL)	−0.601 (−0.825 to −0.216)	0.0039
	Reticulocyte count (%)	−0.656 (−0.848 to −0.313)	0.0012
	Renal HIF1Alpha expression	−0.520 (−0.806 to −0.036)	0.0346
	Plasma HIF1Alpha (pg/mL)	0.684 (0.358 to 0.861)	0.0006

**Table 3:** Multivariate regression analysis of the association between FGF23 and independent variables of interest. Each estimate derives from a separate regression model adjusted for serum phosphorus and creatinine. The table shows the β coefficients, 95% confidence intervals (IC95%), and the respective *p*-values. ln(FGF23), natural logarithm of FGF23; CI, confidence interval; HIF, hypoxia-inducible factor.

Independent Variable	Parameter	Standardized β Coefficient (95% CI)	*p*-Value
ln(FGF23)	Hemoglobin (g/dL)	−0.602 (−1.009 to −0.195)	0.006
	Hematocrit (%)	−0.609 (−1.187 to −0.030)	0.040
	Serum iron (mg/dL)	−0.217 (−0.600 to 0.166)	0.249
	Hepcidin (pg/mL)	0.084 (−0.457 to 0.626)	0.746
	Erythropoietin (pg/mL)	−0.142 (−1.011 to 0.727)	0.730
	Plasma HIF1Alpha (pg/mL)	0.543 (−0.078 to 1.163)	0.082
	Renal HIF1Alpha expression	−0.207 (−0.693 to 0.279)	0.512
	Bone marrow HIF1Alpha expression	0.518 (0.111 to 0.924)	0.016
	Renal HIF2Alpha expression	0.149 (−0.268 to 0.567)	0.567
	Bone marrow HIF2Alpha expression	−0.178 (−0.593 to 0.237)	0.378

**Table 4:** Parameters of mineral metabolism in normal rats and in animals with chronic kidney disease receiving or not receiving AntiFGF23. FGF23 was not measurable in the AntiFGF23 experimental group due to antibody interference. Data are expressed as mean ± SD. Initial/final n per group: control (n = 5/5), Nx1.2P (n = 5/4), and Nx1.2P+AntiFGF23 (n = 6/4). Animal losses were due to postsurgical complications following nephrectomy. *, significantly different vs. control; ^#^, significantly different vs. Nx1.2P. Symbols may appear in combination when a value differs significantly from more than one reference group. Nx: nephrectomy; FGF23: intact fibroblast growth factor 23.

	Control	Nx1.2P	Nx1.2P+AntiFGF23
Plasma calcium (mg/dL)	10.91 ± 1.10	10.19 ± 3.38	7.27 ± 2.65
Plasma phosphorus (mg/dL)	3.87 ± 0.72	6.66 ± 1.29 *	14.67 ± 3.41 *^#^
Plasma creatinine (mg/dL)	0.63 ± 0.10	1.10 ± 0.18 *	2.01 ± 0.19 *^#^
Plasma FGF23 (pg/mL)	367 ± 15	6632 ± 385 *	-
Urine calcium (mg/24 h)	0.59 ± 0.28	1.58 ± 0.25	19.30 ± 6.18 *^#^
Urine phosphorus (mg/24 h)	43.95 ± 11.40	164.80 ± 44.72 *	4.17 ± 0.004 ^#^

**Table 5:** Parameters of mineral metabolism in animals with normal renal function receiving either high concentrations of FGF23 or subjected to FGF23 blockade. FGF23 was not measurable in the AntiFGF23 experimental group due to antibody interference. Data are expressed as mean ± SD. Initial/final n per group: control (n = 5/5), FGF23 (n = 5/5), and AntiFGF23 (n = 2/2). Given the small sample size in the AntiFGF23 group, data involving this arm are only descriptive. *, significantly different vs. control; FGF23, fibroblast growth factor 23.

	Control	FGF23	AntiFGF23
Plasma calcium (mg/dL)	8.34 ± 2.24	5.74 ± 3.03	7.92 ± 0.66
Plasma phosphorus (mg/dL)	6.30 ± 0.47	4.76 ± 0.46 *	5.30 ± 0.53
Plasma creatinine (mg/dL)	0.63 ± 0.08	0.56 ± 0.06	0.54 ± 0.06
Plasma FGF23 (pg/mL)	248 ± 48	336 ± 63 *	-
Urine calcium (mg/24 h)	0.20 ± 0.08	0.10 ± 0.07	0.28 ± 0.04
Urine phosphorus (mg/24 h)	20.79 ± 6.42	22.75 ± 11.75	12.28 ± 3.12

## Data Availability

The original contributions presented in this study are included in the article/Supplementary Materials. Further inquiries can be directed to the corresponding authors.

## Supplementary Materials

The following supporting information can be downloaded at: https://www.mdpi.com/article/10.3390/ijms27198618/s1.
