# Supplementary material for: Targeting FGF23 to Ameliorate Anemia in Chronic Kidney Disease
Source: Int J Mol Sci. 2026 Sep 26;27(19):8618. doi: 10.3390/ijms27198618 (PMC13665252; doi:10.3390/ijms27198618)
Supplement: Supplementary file 1 [file ijms-27-08618-s001.zip › ijms-4533443-supplementary.pdf]

SUPPLEMENTARY Figure S1

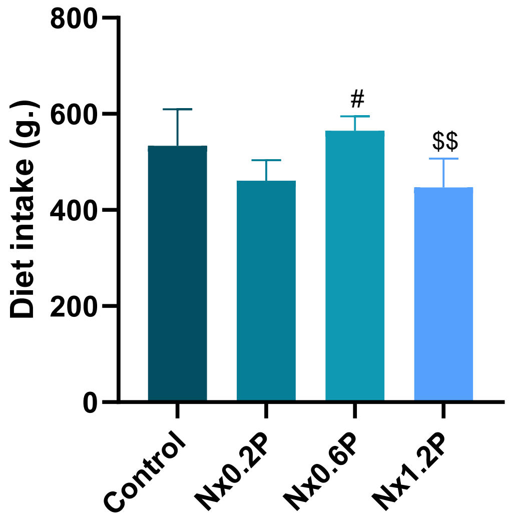

#P<0.05 vs. Nx0.2P
$$P<0.01 vs. Nx0.6P

SUPPLEMENTARY Figure S2

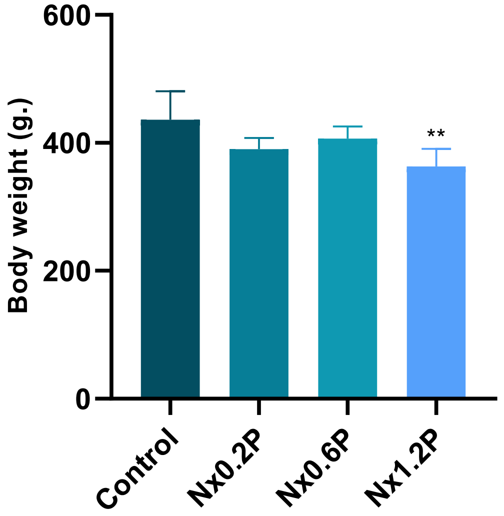

**P<0.01 vs. Control

SUPPLEMENTARY Figure S3

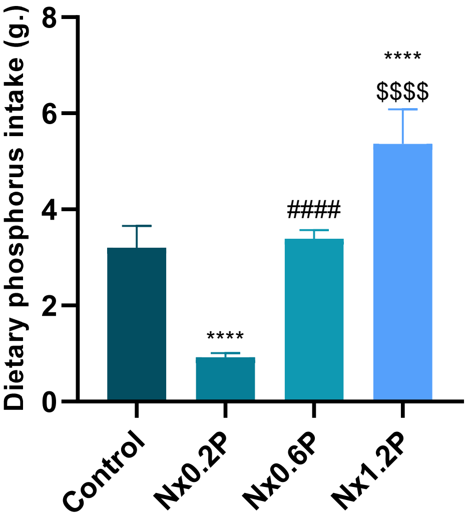

****P<0.0001 vs. Control
####P<0.0001 vs. Nx0.2P
$$$$P<0.0001 vs. Nx0.6P
